# Supplementary material for: Are carbon emissions trading and green financial instruments synergistic? -Comprehensive quantitative research based on content analysis
Source: PLoS One. 2024 Mar 7;19(3):e0298601. doi: 10.1371/journal.pone.0298601 (PMC10919601; doi:10.1371/journal.pone.0298601)
Supplement: S1 Data — (ZIP) [file pone.0298601.s001.zip › Data/地级市Z_F工作报告.zip!/╔╜╬≈/╠1⁄2╘¡╩╨/└┤╫╘╣1⁄2╓┌║┼í╛┬φ┐╦╩2╛▌═°í┐_2009.docx]

各位代表：

现在，我代表市政府向大会报告工作，请予审议，并请各位政协委员和列席人员提出意见。

一、2008年政府工作回顾

2008年是我市各项工作积极推进、破浪前行的一年。全市上下在省委、省政府和市委的正确领导下，坚持以科学发展观为指导，团结一致，开拓进取，经济社会总体上沿着又好又快轨道平稳发展。全年完成地区生产总值1468.1亿元，增长8.1%；规模以上工业增加值581.1亿元，增长2.7%；财政总收入306.9亿元，增长27.7%；一般预算收入116.9亿元，增长32.2%；全社会固定资产投资额702.6亿元，增长21.8%；社会消费品零售总额619.9亿元，增长20.2%；城市居民人均可支配收入15230元，增长10.8%；农民人均纯收入6355元，增长14.3%。市政府向全市人民承诺的十件实事全部超额完成！

（一）经济转型步伐加快，三大产业总体保持平稳发展。

工业绿色转型加快推进。不锈钢园区、汽车工业园、铝镁合金基地建设加紧推进，太重煤机成套设备项目建成投产。规模以上工业万元增加值能耗下降13.7%。7家单位列入省循环经济试点。工业经济效益综合指数达到194.6，比上年提高5.6点。高新区、经济区、民营区工业总产值分别增长19%、23.6%、15%。民营经济增加值完成680亿元，增长16.9%；上缴税金160亿元，增长53.2%。

农村经济持续向好。农村经济总收入551.6亿元，增长10.7%。绿色农业和品牌农业加快发展，认证无公害农产品63个。国家级重点农业产业化企业达到4家、省级24家。农民专业合作社规范发展到583个。实施扶贫项目120多个，2.7万人脱贫。土地开发整理1.6万亩。建成大型秸秆供气工程14座。农村公路新改建1472.2公里。治理水土流失11.5万亩。解决了11万农村人口饮水安全问题。

第三产业快速增长。服务业增加值完成706亿元，增长13.3%。汾东现代服务业新区和太原滨西商务中心等重大项目开工建设。大力实施“万村千乡市场工程”、绿色便民早餐工程，建设新农村便利店117个，新建、改建农家店327家。大力开展“零售业节能行动”，认真贯彻商务部“限塑令”。实施晋祠环境综合整治二期、天龙山景区改造等工程。蒙山大佛景区成为旅游市场的新亮点。实现旅游总收入165.7亿元，增长24.7%。

（二）城市建设与管理不断加强，城乡面貌持续改善。

基础设施建设取得重要进展。组织编制2008—2020年城市总体规划纲要，编制完成公共交通、住房建设等一批重点规划。完成投资57.8亿元，实施基础设施工程项目76项，新建改建道路78.1公里、桥梁11座。迎泽西大街改造等一批主次干道改造项目竣工通车，柳巷南北路等33条小街巷完成改造，汾河美化南延等建设项目进展顺利。房地产开发投资121.6亿元，增长28.2%。城市居民棚户区改造、经济适用住房、廉租住房建设加快推进。城中村改造和村镇建设稳步展开，建成村民安置住宅64万平方米，新开工重点小城镇建设项目40个。

城市管理水平不断提高。实现国土纯收益31.2亿元。关闭自备井131眼。天然气置换23万户。集中供热扩网551万平方米。坚决打击违法用地和违法建设行为，拆除各类建筑182.2万平方米。道路大修和小修碎补27万平方米。深入治理车辆超限超载，超限超载率从治超前的13%下降为0.09%。完成数字城管中心及城区二级指挥中心建设，并在107平方公里范围内试运行。全面开展城乡清洁工程，累计清理卫生死角1.3万处，清除积存垃圾745万方，取缔无照经营摊点7.8万个，城乡面貌逐步改善。

（三）绿色标准制定与实施工作进展良好，生态环境保护与治理不断加强。

绿色标准制定和“绿色十佳”创建工作取得突破。发布《太原市绿色工业企业管理导则》、《太原市绿色学校管理规范》等15个绿色标准，初步形成绿色转型标准体系的重点框架，我市成为拥有绿色地方标准最多的省会城市，太原的科学发展标准化工程取得重要突破。制定《太原市绿色转型促进条例》并通过省人大批准，我市成为全国第一个制定实施整体推进绿色转型地方法规的省会城市。稳步推进“绿色十佳”创建，确定重点创建目标单位300多家，为全方位、深层次加速绿色转型打下初步基础。

生态建设和环境治理加快推进。汾河治理修复和西山地区综合整治正式启动。植树造林20.4万亩。完成汾河湿地公园北延工程。市区绿化覆盖率、绿地率均新增1个百分点，人均公共绿地新增0.61平方米。严格执行绿色高压线，对76个建设项目予以否决，关停焦化项目21个，取缔淘汰高污染企业226个，炸毁无证私开坑口247个。90座9万吨以下煤矿关闭重组工作全部完成。新增9平方公里高污染燃料禁燃区。投入17亿元治理工业污染，污染物减排任务全部完成。市区空气质量二级以上天数达到302天，比上年增加33天。

（四）改革开放迈出新步伐，自主创新能力和科技实力不断提升。

重点领域改革不断深化。颁布投资项目管理办法。出台煤炭可持续发展基金安排使用管理办法。太原市商业银行重组更名为晋商银行。全力推进国企改革，市属工业、商贸企业已改制及正在改制的企业达到96.4%。积极稳妥地安置破产企业职工，为8363名职工解决了工资、医疗等拖欠费用。优化国有经济布局，南方重汽、无缝钢管制造工业园等项目建设加快推进。

对外开放进一步扩大。积极组团参加“煤博会”、“中博会”等重大经贸洽谈活动。新批设立外商及港澳台商投资企业27户。实际利用外资3.12亿美元，增长31%。到位境内省外资金89.2亿元，增长20.3%。成功举办欧盟—中国山西地区企业合作项目洽谈对接会、中国（太原）镁及镁合金产业高端论坛。地区外贸进出口总额实现93.9亿美元，增长15.8%。

科技实力不断增强。大力开展“技术创新攻坚年”活动。规模以上工业企业拥有自主知识产权达到25%。积极创建国家知识产权示范城市，专利申请量3012件，增长68%。21项科技成果达到国际领先或先进水平，9项获得国家科技奖，首获省科技发明一等奖，科技成果转化率达到43%。高分子材料实验室成为我市首家国家实验室，科伟通孵化器成为我市首家国家级科技企业孵化器。我市跻身中国城市综合创新能力50强。

（五）重大民生问题逐步解决，各项社会事业取得较快进展。

民生问题得到进一步解决。全面落实积极的就业政策，全市城镇新增就业13.9万人，下岗失业人员再就业5.15万人，城镇登记失业率控制在3.3%。强化劳动执法维权，追回被拖欠工资3843万元。社会保险参保人数继续增加，城镇社保基金覆盖率达到86.5%。在全省率先启动居民医保工作，提高了企业退休人员待遇、城镇失业保险标准和企业最低工资标准。城市低保标准、人均补差和农村低保覆盖率均居全省首位。新建改造农村敬老院35所，在全国率先实现农村五保户全部集中供养。慈善总会救助城乡贫困人群20余万人。合理使用价格调节基金，建立“物价上涨与困难家庭临时生活补贴联动机制”。

教育事业实现新突破。加快实施“百校兴学”工程，规划新建21所、改扩建81所学校，总建筑面积50余万平方米，总投资10.1亿元。免除全市46万名义务教育阶段学生学杂费。规范“小升初”工作，改革中考招生政策。推进义务教育标准化建设，已有8个县区通过省义务教育标准化达标验收。扩大优质高中教育资源，9所学校通过省级示范高中验收。

卫生事业取得新成果。出色完成结石患儿筛查救治、国际性会议赛事的卫生应急保障任务。巩固新型农村合作医疗制度，参合率达到94.2%；市本级财政补助1211万元，参合率和补助标准均居全省第一。县乡村三级卫生机构达标率78.4%。实施卫生惠民工程，完善农村巡回医疗和支农专家组工作制度，认真落实济困门诊等缓解群众“看病难、看病贵”的措施。坚持科教兴医，2项科研成果达到国际先进水平，3项达到国内领先水平。

体育事业创历史佳绩。成功举办2008国际自行车联盟小轮车世界锦标赛。在第十八届全国“飞跃”杯田径比赛上，获得团体第二的好成绩。省运会阶段赛成绩优良，获得156枚金牌。“全民健身与奥运同行”等群众体育活动蓬勃开展，体育场馆设施建设加快进行。

人口自然增长率得到有效控制，出生人口素质进一步提高。外事、侨台、民族、宗教、人防、气象、地震、档案、地方志、国防后备力量建设等工作和老龄、妇女、残疾人、红十字、关心下一代、革命老区建设等事业都取得了新成绩。

（六）文化建设与文明创建加快推进，社会和谐稳定与民主法制建设不断加强。

特色文化名城建设加快推进。正式启动国家历史文化名城申报工作。完成古建筑、古村落、古树古木三项普查。启动湖滨会堂改扩建和市美术馆、博物馆等一批文化建设工程。成立了由12家企业组成的创意产业联盟。加快基层文化设施建设，14个文化广场建成使用。推出晋剧《龙兴晋阳》等一批优秀作品，荣获国家级奖12项。太原锣鼓等9个项目列入第二批国家级非物质文化遗产名录，晋剧等3个项目列入第一批扩展名录。晋商文化艺术周荣获“2008年度中国十佳文化节庆”称号，“晋阳之春”文化节、中华傅山园祈福庙会等活动效果良好。

文明创建活动深入开展。连续三次获得“全国文明城市创建工作先进城市”荣誉。在全国首次评选中，被中央文明委授予 “全国未成年人思想道德建设先进城市”称号。再次获得“全国双拥模范城”称号，实现“六连冠”。

社会保持和谐稳定。“两抢一盗”警情下降68%，群众安全感和满意度不断提升。深入开展矛盾纠纷排查化解工作，依法妥善处置群体性事件。全面落实安全生产责任制，大力开展“安全生产隐患治理年”活动，安全生产形势保持稳定。扎实推进地质灾害防治，组织应急避险搬迁794户。建立健全食品安全长效机制，严把食品市场准入关、检测关、退市关。全力清查整顿乳制品生产企业和市场。积极解决信访突出问题，信访形势持续好转。

民主法制建设不断加强。各级政府自觉接受人大监督，大力支持政协履行职能，认真接受民主监督。人大代表建议和政协委员提案答复率100%。围绕全市中心工作，认真听取各民主党派、无党派人士和群众团体的意见。积极开展法制宣传和法律援助，全民法制观念逐步增强。圆满完成第八届村委会换届选举工作。强化行政监察和审计监督，政府自身建设得到加强，廉政建设取得新成效。

（七）服务大局，为抗震救灾和北京奥运做出贡献。

大力支援四川抗震救灾。全市向地震灾区捐款捐物1.97亿元。派出12批医疗救援队伍参与抗震救灾。援川特警被公安部授予“灾区群众满意的公安特警队”荣誉称号，获集体一等功。成立太原援川建房突击队，奔赴四川地震灾区建设过渡安置房7408套、14.1万平方米，提前两个月全面、超额完成援川建房总任务。援川建房太原突击队被中共中央、国务院、中央军委授予“全国抗震救灾英雄集体”光荣称号，被中华全国总工会授予“全国五一劳动奖状”。

全力做好北京奥运相关工作。圆满完成奥运火炬太原接力活动，北京奥组委授予我市“奥运火炬传递突出贡献奖”。强化奥运安保工作，深入开展“晋阳风暴”专项行动，确保了奥运火炬传递等一系列重大活动的安全。

各位代表，回顾不平凡的2008年，深感成绩来之不易。这是省委、省政府和市委正确领导的结果，是市人大、市政协和各位代表、各位委员监督支持的结果，是全市人民团结奋斗的结果。在此，我代表市政府，向辛勤工作在各条战线的工人、农民、知识分子和各级干部，向大力支持政府工作的人大代表、政协委员、各民主党派、工商联、人民团体、无党派人士，向关心帮助太原发展的国家、省驻并单位和驻并部队、武警官兵，向积极参与太原建设的港澳台同胞、海外侨胞和国际友人，表示衷心的感谢！

在肯定成绩的同时，还必须清醒地看到，我市经济社会发展还存在很多困难和问题：资源型、高消耗产业仍占主要地位，绿色工业体系建设任务较重；实现节能减排目标、治理和保护生态环境压力较大，推进绿色转型任重道远；“城市病”日益突出，城市规划、建设、管理面临不少矛盾和问题；现代服务业和文化产业比重偏低，发展水平不高；社会事业发展欠账仍然较多，城乡居民就业、医疗、教育和社会保障等问题还要下力气解决；安全生产形势不容乐观，经济社会发展中的不确定因素增加，影响社会和谐的因素还有不少；体制机制创新深化不够，政府职能需要加快转变。对于这些问题，我们一定采取有力措施，在今后工作中努力加以解决。

二、2009年政府工作的指导思想和奋斗目标

2009年是推动“十一五”规划全面实施的重要年份，更是应对危机挑战、扩大内需、确保平稳较快发展的关键一年。2009年政府工作的指导思想是：以邓小平理论和“三个代表”重要思想为指导，深入学习实践科学发展观，紧紧抓住国家扩大内需的重大机遇，积极应对国际金融危机的严峻挑战，围绕建设新型工业基地、特色文化名城、现代宜居城市的目标，坚持创新型城市建设主战略，深入推进绿色转型，以八个功能区建设为总抓手，以优质项目群建设为载体，加大投资保增长，以人为本保民生，构建和谐保稳定，调整结构促转型，深化改革增活力，坚定信心，迎难而上，全力推进三城联动发展、城乡统筹发展、安全和谐发展。

主要预期目标是：地区生产总值增长8%左右；工业增加值增长6%；全社会固定资产投资增长25%；社会消费品零售总额增长15%；财政总收入、一般预算收入均增长8%；城镇居民人均可支配收入、农民人均纯收入均增长9%；居民消费价格总水平涨幅控制在4%以内。以上是导向性指标，根据形势变化可以适度调整。坚决完成以下约束性指标：万元地区生产总值能耗下降5.2%；二氧化硫、化学需氧量继续保持下降，确保完成省政府下达的减排任务；城镇新增就业人数10万人，城镇登记失业率控制在4.2%以内；亿元地区生产总值安全生产事故死亡人数等安全生产指标严格控制在省核定的控制目标之内。

根据上述指导思想和预期目标，今年政府工作的中心任务是坚决抓住国家扩大内需的重大机遇，积极应对金融危机的挑战，坚决把发展的重心转移到创新转型、优化结构、打好基础上来，着力破解多年来制约我市发展的难点、瓶颈和短板问题。总体上坚持“四个优先”、突出“六个加快”，即在经济社会发展中坚持民生优先、生态优先、科教优先、安全优先；加快八大功能区和宜居城乡建设，加快绿色产业发展，加快生态建设和基础设施建设，加快体制机制创新，加快持续投资能力建设，加快“五条社会保障线”建设，在科学发展道路上迈出坚实步伐。

三、2009年政府工作的主要任务

2009年，要在统筹兼顾、全面推进的基础上，重点做好以下八个方面的工作：

（一）全力抓好优质项目群建设，千方百计扩大投资，确保经济平稳较快发展。

抓好优质项目群建设。牢牢抓住当前大上项目、低成本上项目的重要机遇期，把应对国际金融危机、深入推进绿色发展的措施落实到项目，具体到产品，全面加速我市已经确定的产业、城乡、生态、民生等方面的优质项目群建设。强化项目领导责任制，认真落实好配套资金，加大监督、管理和服务力度，加快推进中央和省重点项目的落实。加大项目编报和争取力度，力争有更多项目列入中央和省里的计划。站在更高起点规划产业发展，在原有基础上，争取用三到五年时间，培育出100个产值在10亿元至100亿元以上的“绿色百强”企业。

千方百计扩大投资规模。以项目为载体，全力扩大投资规模。按照“政府投资、市场融资、调动民资、招商引资”多渠道筹集资金的思路，建立和完善财政资金为引导，银行贷款、资本市场等投融资方式为主体的“大融资”平台，加大对重点工程、民生工程、支柱产业和中小企业发展的金融支持力度。用好用足国家支持循环经济、节能减排、高新技术、生态建设等财税金融政策，以规费减免、信贷倾斜等措施推进重点项目建设。

（二）加速推动产业绿色转型，努力构建充满活力、富有竞争力的产业发展格局。

加快绿色工业体系建设。突出抓好210个重点项目的开工建设和投产达产，培育载重汽车及零部件、煤机成套设备、铁路装备、风力发电装备、清洁能源、镁及镁合金等六大产业集群。着力加快建设铁路装备工业园，打造全国重要的铁路装备制造新基地。推进企业自主创新能力建设，加快突破关键技术。强化工业经济运行监测，提高工业经济运行质量。注重运力、电力等生产要素的综合协调和保供，千方百计缓解企业资金压力，全力帮助企业走出困境。

推动现代农业快速发展。统筹推进农村土地、集体林权和户籍、金融等各项改革，积极推进农村土地承包经营权流转。完善绿色种养业生产标准，构建绿色农业产业体系。围绕农民增收，重点发展一批都市型绿色农业项目，新建5个万间绿色日光节能温室群，形成我市无公害蔬菜生产核心区。以建设高效生态农业示范基地为目标，高标准建设10个无公害农产品基地。突出抓好健康养殖业，建设规模养殖园区20个。全力推进农业产业化经营，增强龙头企业的带动能力。积极扶持和引导农民发展专业合作社，不断提高组织化程度。农村经济总收入实现595亿元、增长8%。

促进第三产业上规模、上水平。加快发展以产品设计、研发、人力资源、金融、咨询、外包为重点的生产性服务业。积极发展房地产物业服务、社会化养老服务等新兴服务业。培育一批具有完备物流配送体系的企业，发展为现代物流业配套的城市设施。加大农村市场建设投入，抓好“万村千乡市场工程”，建设3—5个大型农资、农产品批发市场，做好家电下乡工作。以晋祠环境综合整治为切入点，打造晋祠、蒙山等特色文化旅游品牌。以“2009中国生态旅游年”为契机，推出一批生态旅游项目。充分利用动车开通的机遇，构建与京、津、冀双向交流、良性互动的大旅游区发展格局。

充分发挥园区经济的拉动作用。推动各个经济园区“二次创业”。高新区要着力建设创新能力突出、产业特色鲜明的现代科技园区。经济区要大力提升循环经济和产业集群发展水平。民营区要加快工业新区和城东民营现代物流区建设步伐。不锈钢产业园区要加快基础设施建设，确保重点项目早日投产达效。

加速提升壮大非公有制经济。全面实施中小企业成长工程，抓好千户小企业孵化工程、成长性中小企业扶持工程和骨干企业培育三大工程建设。努力破解中小企业融资难题，组建小额贷款公司和担保公司，政府按担保金额落实风险金与补偿金，增强担保机构的担保能力与抗风险能力；积极促进金融机构提高信贷支持力度；支持中小企业在创业板上市；大力推进股权融资。

（三）以八大功能区建设统筹城乡发展，加快绿色城市化和城乡一体化进程。

切实推进八大功能区建设。围绕西山创意产业、文化旅游区，汾东高新产业、现代服务业区，城东民营现代物流区，北部不锈钢生态工业区，古交新型煤化工及工补农示范区，清徐汾河高效观光农业区，阳曲新型工业承接区和娄烦生态旅游经济区八大功能区的定位，打破行政区划，完善功能区规划，在基础设施建设、节能减排与生态建设、自主创新和产业结构调整等方面，加快谋划和筛选一批新上项目，推出切实可行的政策措施，为我市绿色城市化和城乡一体化发展开拓新的广阔空间。各县区要按照八大功能区建设要求，进一步优化经济布局，大力推进县域经济科学发展、特色发展。

全面推进西山生态功能区建设。围绕打造“绿色西山、活力西山、和谐西山、人文西山”的目标，大力推进西山地区综合整治。全面启动并加快西山地区企业搬迁步伐，对不符合国家产业政策、区域功能规划以及安全生产和环保要求的企业，坚决依法予以关停。加快长风西大街打通、西山货运专线等基础设施建设。加快太原铁路装备工业园以及物流、文化创意等接替产业发展。实施西山地区地质灾害治理工程，推进以万亩稻田为重点的西山湿地建设。完成西环高速两侧、万亩生态园二期和蒙山绿化等一批绿化工程。力争用3—5年时间，把西山地区建设成为生态环保、绿色宜居、人地和谐、山川秀美的现代化新城区。

加快城市基础设施建设。围绕“三城联动”发展，力争实现市区范围内的控规全覆盖，突出抓好“一河两岸”、重点片区、重点专项的规划编制。加快重点项目建设进度，高质量建设好103项重点工程。抓好滨河东西路南北延、旧晋祠路改造等主次干道的新建、改造，完成祥云桥等5座桥梁及2个高速互通立交桥的建设。打通府东街、千峰北路等断头路。对66条背街小巷进行改造提档。全力支持山西大医院等省城重点工程建设。编制完成综合交通规划。做好尖草坪—小店—榆次轨道交通线的规划设计等工作。研究规划城市地下综合管网系统改造工程。加快重点片区工程进度，认真抓好晋祠景区二期、龙城新区、龙潭片区等重点项目建设。抓好城西热源厂等项目建设，集中供热扩网1000万平方米。自来水表改造5万户，天然气置换20万户。加快3个污水处理厂的改造升级，提高中水回用率。

加大新农村建设力度。对新农村建设试点村和重点推进村进行科学指导，实现县、乡、村三级规划的对接。加快太古路改造、西山旅游及防火循环公路一期等重点工程建设，实施县乡公路改造、村村通水泥（油）路、公路安全保障与危桥改造三项工程。高标准推进“四化四改”，搞好“百镇（乡）千村万户”生态示范工程和生态沼气建设工程，注重发展秸秆气化站和太阳能等农村新能源。继续抓好农村劳动力转移就业、产业开发、整村推进、移民并村、老区建设等重点工作，改善贫困地区农民的生产生活条件。加大城中村改造力度，规范改造行为，力争整村改造取得突破。抓好小城镇规划与建设。做好“山西省历史文化名镇（名村）”的申报工作，加强对历史文化名镇及古村落的保护和利用。

强化城乡长效管理。继续推进城市基础设施管养机制改革，建立按养护作业质量和数量核拨经费的机制，完善基础设施养管绩效考核机制。充分发挥城市存量资产的效用，提高市场化运作水平。加强土地市场动态监管与监测，完成国土收益20亿元。继续治理超限超载。推进数字化城市管理高位监督和指挥组织体系建设，统筹和优化配置全市信息资源。优化公交线网，适当扩大出租车规模。加大执法力度，切实解决供热收费、城市养犬、鞭炮燃放等群众反映较多的热点问题。严厉打击违法违章建设，实现全年新建工程零违法的目标。深入推进城乡清洁工程，进一步提高城乡清洁水平。

（四）深入实施科学发展标准化工程，加快资源节约型和环境友好型社会建设。

狠抓绿色标准实施和“绿色十佳”创建工作。加快出台具体实施办法和工作方案，认真贯彻《太原市绿色转型促进条例》。全方位开展绿色标准实施工作，把绿色标准制定和实施工作纳入各部门、各县区的常规工作中，积极制定具体的政策措施，强化监督考核。以“绿色十佳”创建为载体，扩大绿色标准的实施覆盖范围。把产业领域的创建活动与实施品牌战略结合起来，提高品牌知名度与竞争力。继续制定和完善绿色标准，积极争取国家循环经济标准化试点市政策。

大力发展循环经济，促进节能减排。认真落实国家能耗限额标准，对全市企业用能、节能情况进行调查摸底。对能耗3000吨以上的重点用能企业实施能源审计。对列入省、市重点节能规划的22个项目，要加快进度，争取年内完工。实行项目节能评估审查制度，严格准入条件。年内全面完成重点行业20个企业的强制性清洁生产审核。组织实施好城市污水综合利用等循环经济示范项目。支持清徐、古交建设循环经济示范县（市）。加强再生资源回收利用网络体系建设，推广可再生能源利用技术。新建建筑严格执行50%节能标准。

强制性淘汰落后产能。按照“绿色高压线”要求，把好项目准入关。妥善做好煤矿兼并重组和整顿关闭工作，今年再关闭重组煤矿80座。整顿规范矿产资源开发秩序，全面完成非煤矿山资源整合和有偿使用，压减矿山25%。按照国家产业政策，严格执行“三个不批”、“三个限批”，切实巩固土小企业及落后生产能力关停淘汰成果，严厉打击土小企业“死灰复燃”。

强力推进环境保护和生态建设。以实施“蓝天碧水工程”为龙头，抓好24项环保限期治理工程。大力实施“煤改气”工程，加强城中村燃煤污染控制，拆除土小燃煤锅炉5000台以上。严格控制扬尘污染。汽车尾气环保检验合格率达到70%。工业固废综合利用率达到50%以上。积极推进汾河流域生态治理及修复保护，汾河两侧3公里范围内实现废水“零排放”。空气质量二级以上天数保证完成省定任务。严格落实占用耕地“占补平衡”、“先补后占”。完善环城林带提档增绿工程，造林20万亩。积极创建国家园林城市，重点抓好学府公园等39项园林绿化工程。加速推进中心区100个小游园建设。高标准完成汾河公园二期南延等续建工程，启动太山植物园、动物园周边整治等项目。着手对晋阳湖实施整治。绿化覆盖率、绿地率提高1个百分点，人均公共绿地净增0.5平方米。

（五）高度关注民生和安全稳定，加快发展社会事业，提升和谐发展水平。

以构建义务教育、医疗、就业、住房、环境管理“五条社会保障线”为中心，着力保障和改善民生，确保安全稳定，在和谐社会建设上迈出新的步伐。

千方百计做好就业工作。积极开展创建创业型城市试点工作。多渠道开发就业岗位，统筹做好城镇新增劳动力、下岗失业人员、农村富余劳动力、高校毕业生和残疾人就业。实施积极的就业政策，完善规费减免、小额贷款、社保补贴、就业服务补贴等就业扶持政策。大力实施创业带动就业工程，加强中小企业创业孵化基地建设，支持毕业生自主创业。健全市场信息服务体系，完善四级公共就业服务体系。建立就业困难人员就业援助的长效机制，对下岗失业者和农民工进行特别培训。实行企业裁员报告制度，做好企业劳动用工监测和公共职介市场监测工作。建立健全工资支付监控和保障制度，严厉打击非法用工行为，维护劳动者合法权益。

完善社会保障体系。扩大社会保险覆盖面，加强社保基金监管，全面完成征缴、扩面和审批发放工作。深入落实社会保障惠民政策，提高城镇职工、居民医疗保险待遇和企业退休人员基本养老金水平，被征地农民都要按规定参加社会养老保险。积极推进新型农村养老保险试点工作。全面推进城乡一体化的低保救助、医疗救助体系建设。整合优抚抚恤、救灾救济、医疗救助、五保供养、社会福利等优待救助政策，全力保障困难群体的基本生活。充分发挥红十字会、慈善总会的作用，扎实开展慈善救助活动。

加快保障性住房建设。出台促进房地产业持续健康发展的政策措施，拉动住房消费。大力推进城市居民棚户区改造、经济适用房和廉租住房建设。抓好农村困难群众住房建设。完善住房公积金的缴收管理。加大监管力度，严厉查处房屋拆迁方面的违法违规行为。规范市场行为，保护公平竞争，有效改善全市人民的居住条件。

推进教育事业迈上新台阶。加快“百校兴学”工程建设。重点改建存在较大安全隐患的学校及农村学校，基本完成中小学校D级危房改造任务。全面实施“校校有标准操场工程”。以提升初中学校办学质量与水平为重点，深入推进义务教育标准化建设。大力发展中等职业教育和技工教育，形成优势专业群。加快太原大学、太原师专建设进程，提高市属高职高专办学实力。积极推进教育人事制度改革。继续做好“两免一补”工作。认真做好进城务工人员子女接受义务教育工作。严禁教师举办或参与举办向学生收费的有偿培训。对普通高中学校招收择校生严格管理，坚决制止各种名目的乱收费。

加快发展卫生事业。积极贯彻国家医改方案，制定配套措施，深化医药卫生体制改革。健全突发公共卫生事件应急管理制度，落实重大疾病防治政策和措施。加强基层医疗卫生服务体系建设，组织城市医院对口支援乡镇卫生院、社区卫生服务中心，提升农村、社区卫生服务水平。将85%的社区卫生服务机构纳入医保，方便市民就医。巩固新型农村合作医疗成果，筹资标准提高到100元/人以上，参合率95%以上，简化报销程序，提高报销比例。深入开展创建国家卫生城市活动，进一步提升城市卫生总体质量。

推进各项事业全面发展。抓紧建设滨河体育场。积极备战全运会，参赛成绩力争有所突破。发展群众体育，开展“五个百万人群”和“两个关爱人群”健身系列活动。稳定低生育水平，提高人口素质。扎实做好第二次全国经济普查工作，确保数据质量。外事、侨台、民族、宗教、气象、人防、文物、档案、地方志等各项工作和老龄、妇女、残疾人、关心下一代等各项事业，都要实现新发展，取得新成绩。

切实维护社会稳定。坚持科技强警，大力推进公安信息化和装备现代化建设。推进公安机关警务机制改革创新，加强执法规范化建设，构建和谐警民关系。引深平安创建活动，继续开展“晋阳风暴”行动，依法严厉打击各类犯罪活动，重点打击群众反映强烈的“两抢一盗”案件，确保发案率有大的降低、破案率有大的突破。建立健全群体性事件处置机制，加强人民调解工作，积极化解矛盾纠纷。严格落实信访责任制，坚持依法接访、常态接访、主动下访，进一步畅通信访渠道。深入排查化解矛盾纠纷，切实解决群众反映强烈的热点难点问题。建立健全应对和处置突发事件的机制，提高应急管理水平。加强防震减灾基础建设，提升综合防御能力。

提高安全发展水平。强化企业和政府两个主体安全生产责任，认真贯彻落实安全生产“十项制度”，切实抓好国家“安全生产年”和省为期一年的安全生产专项整治工作，加强对重大危险源的排查和监控，突出抓好煤矿和非煤矿山安全，对存在安全隐患的尾矿库坚决实施关闭。设立安全生产专项基金，建立安全生产教育培训学校。加快人防工程建设，促进两防一体化发展。加强电网建设和安全供用电管理。确保市政公用行业安全稳定运行。以质量安全、药品安全和食品卫生安全为重点，集中力量进行治理整顿，严防恶性事件的发生。加强粮、油、蛋、肉等重要生活消费品的储备管理，维护市场秩序，保证价格总水平基本稳定。抓好城乡饮水安全工作。实施农产品“放心工程”，加强农产品、畜产品检测和重大动物疫病防控三大体系建设。依法启动蔬菜、果品市场准入制度。

（六）积极推进特色文化名城建设，努力创建全国文明城市。

加快振兴文化产业。扎实做好国家级历史文化名城申报工作，修复建设府城城墙遗址公园等五个重点项目。认真贯彻落实《太原市促进文化产业发展条例》，实施文化产业振兴工程，设立文化产业发展专项资金，壮大文广、演艺、太原日报报业、高新区创意产业、新晋商联盟文化传播“五大文化产业集团”，打造清徐文化产业园区，培育一批文化产业示范基地，支持民营资本发展文化产业。

推进文化事业繁荣发展。深化文化体制改革，全面构建公共文化服务体系。加快东方明珠湖滨广场、太原美术馆、博物馆、北齐徐显秀壁画馆建设。新建、改扩建20个乡镇综合文化站。搞好建国60周年、太原解放60周年系列纪念活动。办好2009年晋商文化艺术周。深入实施文化下乡、农村电影放映、广播电视“村村通”等文化惠民工程。抓好文化信息资源共享工程平台建设。完成非物质文化遗产保护普查。加强广播电视和文化市场监管，净化社会文化环境。

积极创建全国文明城市。充分发挥政府职能部门在创建工作中的表率作用，对照《全国文明城市测评体系》要求，针对薄弱环节，抓好综合整治。广泛开展群众性文明和谐创建活动，不断提升全体市民综合素质的整体水平，不断赋予特色文化名城新的时代内涵，力争尽快进入全国文明城市行列。

（七）扎实推进体制机制创新，提高对外开放水平。

进一步深化国企改革。完成15户破产企业破产终结和企业重组工作。对已基本完成改制的企业进行“回头看”，确保改制规范完善。坚持搬迁改造和企业发展相结合，实现国有资本的调整和国有企业的战略重组。优化资本配置，重点培育和发展重型汽车、锅炉制造、铝材深加工、煤炭深加工、光电子制造等具有比较优势的产业。加快推进县区国有企业改革。

按时完成机构改革任务。高起点谋划、高标准实施、高质量完成新一轮地方政府机构改革任务。加快推进事业单位分类改革，调整事业单位布局，继续推进事业单位试点改革。

深入推进财政体制改革。加大财源建设力度，加强税收征管，搞好税收服务。优化投资结构和财政支出结构，加大财政对重点领域和关键环节的支持力度，坚持财政增量向社会事业、民生事业、困难群众生活领域倾斜。厉行节约行政成本，坚决压缩公务消费，会议费、接待费、购车费等在近三年平均水平基础上压缩15%以上。健全现代财政国库管理制度，规范预算执行。

强化科技支撑与引领作用。开展“民生科技促进年”活动，研发和应用一批能够拉动内需、扩大就业、具有自主知识产权的重大科技项目，促进中小企业提高自主创新能力。组织科技人员深入基层服务企业，积极培育产学研战略联盟和产业集群技术联盟。加大科技投入，确保财政科技经费依法达标。拓宽科技风险投融资渠道，全社会研发经费占地区生产总值比重达到2.1%。全市专利申请量4000件以上，规模以上工业企业拥有自主知识产权占30%以上。经省鉴定的科技成果突破100项，科技成果转化率达到45%。力争再次进入全国科技进步先进市行列。

继续扩大对外开放。科学筛选招商项目，引导外资投向现代服务业、现代农业、高新技术产业、高端制造业。强化对内开放，积极引进符合绿色转型要求的东部转移项目。重点抓好项目对接、签约项目履约和千万美元以上项目的审批与跟踪服务。继续实施出口市场多元化战略，鼓励和支持企业调整产品结构，开拓新兴市场，扩大产品出口。研究出台对出口企业的扶持优惠政策，帮助企业渡过难关。积极推进太原经济圈建设，加快汾东新区建设，在旅游、交通等方面率先取得实质性进展。

（八）加强民主法制建设和政府自身建设，迎难而上抓好落实。

推进民主法制建设。各级政府要定期向人大及其常委会报告工作，自觉接受人大的法律监督和工作监督，主动接受政协的民主监督，优质高效办理人大代表建议和政协委员提案。深入开展普法教育，提高全民法律素质。加强国防后备力量建设，落实优抚安置政策，增进军政军民团结，继续创建“双拥模范城”。

各位代表，应对严峻挑战、抢抓发展机遇、完成艰巨任务，是对政府、对干部队伍综合素质的严峻考验。非常时期需要非常作为。各级政府和领导干部要切实加强自身建设，以良好的精神面貌和扎实的工作作风带领群众攻坚克难、共渡时艰。以深入学习实践科学发展观活动为契机，切实增强科学发展意识，努力在发展思路、工作方法上有所创新；在抓住机遇、化解难题上有所作为；在完善机制、加快发展上多下功夫。着力转变职能、创优服务。继续清理规范行政审批和行政收费，高效履行经济调节、市场监管职能，着力提供更多更好的公共产品和公共服务。坚持依法行政、加强管理。优化政府机构设置，按照法定权限和程序行使职权、履行职责，规范行政行为，提升决策的科学化、民主化水平；认真实行政府绩效管理和行政问责制度，提高政府公信力。大力改进作风、狠抓落实。牢固树立科学的政绩观，把更多时间放在深入基层、调查研究、解决问题上；着力提高政府执行力，加大对重点工作、重点项目和重点目标的督促检查力度，建立健全、高效运行“三位一体”的督查机制。坚持从严治吏、廉政勤政。严格执行反腐倡廉的各项规定，全面落实党风廉政建设责任制，强化审计监督，提高廉洁自律能力。各级各部门都要紧紧围绕自身职责和工作目标，自加压力，奋发进取，在全市上下创造想干事、能干事、干成事的浓厚氛围，努力开创各项工作新局面。

各位代表，越是在困难的时候，越要关注民情、化解民忧、改善民生，高度重视困难群众的生产生活，帮助他们解决实际困难和问题！今年市政府承诺办好以下十件实事：一是深入推进“百校兴学”，新建、改建、扩建112所学校，维护、加固和改造中小学校舍27.6万平方米以上。二是实现县乡医疗卫生机构基础设施达标率80%，卫生室空白村卫生室建成率80%。三是建成市劳动保障服务中心，城镇居民基本医疗保险覆盖40万人，农村社会养老保险试点覆盖25万人，6.1万农村特困群众纳入低保范围。四是解决10115名重点优抚对象的就医问题，对4500名残疾人实施康复救助，为100个社区（村）配发康复训练器材。五是建设太原市慈善职业技术学校，帮助1.07万名城镇就业困难人员实现就业，农民技能培训2万人，转移农村劳动力2万人。六是建设农村公路通畅工程100公里，改造农村公路480公里。七是解决农村10万人口饮水安全问题，在全省率先实现农村安全饮水全覆盖。八是发展连锁农家店和新农村便民店100家、放心粮油店150家。九是实施旅游惠民工程，推出旅游“一卡通”、“一证游”服务。十是抓好保障性住房建设，开工建设经济适用住房14.3万平方米，城市居民棚户区19万平方米，廉租住房4.2万平方米，为260户农村贫困残疾人家庭实施危房改造。

各位代表！让我们在科学发展观的指引下，按照省委、省政府的决策部署，在中共太原市委的坚强领导下，同心同德，攻坚克难，圆满完成今年的各项任务，在率先科学发展的征程中迈出更加坚实的步伐，以优异的成绩向新中国成立60周年献礼！
